# Motoric Cognitive Risk Syndrome: prevalence and associated sociodemographic and clinical factors in primary care settings in Brazil

**DOI:** 10.1002/alz70861_108469

**Published:** 2025-12-23

**Authors:** Lilian Viana dos Santos Azevedo, Alice Moreira dos Santos Marques, Geraldo Alves Silva, Giselle Stephanie Santos, Laura Pereira Faria, Maria Luíza Taveira, Marina Nascimento, Maira Tonidandel Barbosa, Paulo Caramelli

**Affiliations:** ^1^ Universidade Federal de Minas Gerais, Belo Horizonte, Minas Gerais Brazil; ^2^ Universidade Federal do Rio de Janeiro, Rio de Janeiro, Rio de Janeiro Brazil; ^3^ Faculdade Ciências Médicas de Minas Gerais, Belo Horizonte, Minas Gerais Brazil

## Abstract

**Background:**

Motoric Cognitive Risk Syndrome (MCR) is a pre‐dementia syndrome characterized by subjective memory complaints and slow gait speed. MCR is associated with increased risk of dementia and mortality. Studies on MCR remain scarce in low‐ and middle‐income countries, particularly in Latin America. The aim of the present study was to investigate the prevalence of MCR in a community‐sample in Brazil and its associations with sociodemographic and clinical factors.

**Method:**

Cross‐sectional study conducted in four public health/primary care settings in Belo Horizonte, Brazil. Sample size calculation indicated that 310 subjects aged 60+ years should be included. Sociodemographic, clinical, motor (Unified Parkinson’s Disease Rating Scale‐motor; UPDRSm) and cognitive (Addenbrooke’ Cognitive Examination‐revised; ACE‐R) aspects were assessed. Gait evaluation was performed using the APDM Mobility Lab™ system.

**Result:**

The final sample consisted of 355 individuals, with mean age of 71.4 years, 69.6% women. Prevalence of MCR was 11.3%. Univariate analysis showed that MCR diagnosis was associated with older age (OR 2.5, CI 1.1–5.9), lower education (OR 0.92, 95% CI 0.85–0.99), physical inactivity (OR 8.2, CI 3.1–21.5), history of falls in the past year (OR 24.5, CI 9.8–61.0), polypharmacy (OR 7.4, CI 3.4–16.1), depression (OR 2.9, CI 1.5–5.7), previous stroke (OR 4.3, CI 1.4–13.4), poor performance on ACE‐R (OR 0.97, CI 0.95–0.99) and higher scores in subsection III of UPDRS (OR 1.2, CI 1.1–1.3). In multivariate logistic regression analysis, history of falls in the past year (OR 14.4, 95% CI 5.5–37.4), polypharmacy (OR = 4.3, 95% CI 1.8–10.3), and higher scores in UPDRS‐III (OR = 1.1, 95% CI 1.03–1.17) remained significantly associated with MCR.

**Conclusion:**

The present study found a prevalence of 11.3% of MCR among older adults seen at primary care level in Belo Horizonte, Brazil. MCR was associated with polypharmacy, falls and parkinsonian signs. These findings are consistent with previous studies and may provide further insights into this pre‐dementia syndrome, which is easily identifiable at low‐cost at primary care/public health settings.

